# Genomic Insights into the Successful Invasion of the Avian Vampire Fly (*Philornis downsi* ) in the Galápagos Islands

**DOI:** 10.1093/molbev/msaf052

**Published:** 2025-03-28

**Authors:** Aarati Basnet, Catalina Palacios, Hao Meng, Dhruv R Nakhwa, Thomas Farmer, Nishma Dahal, David Anchundia, George E Heimpel, Charlotte E Causton, Jennifer A H Koop, Sangeet Lamichhaney

**Affiliations:** Department of Biological Sciences, Kent State University, Kent, OH, USA; Department of Biological Sciences, Kent State University, Kent, OH, USA; School of Life Sciences, Inner Mongolia University, Hohhot 010070, China; Department of Biological Sciences, Kent State University, Kent, OH, USA; Department of Biological Sciences, Kent State University, Kent, OH, USA; Biotechnology Division, CSIR-Institute of Himalayan Bioresource Technology, Palampur, Himachal Pradesh, India; Charles Darwin Research Station, Charles Darwin Foundation, Santa Cruz, Galápagos, Ecuador; Department of Entomology, University of Minnesota, St. Paul, MN, USA; National Institute of Biodiversity (INABIO), Quito, Ecuador; Charles Darwin Research Station, Charles Darwin Foundation, Santa Cruz, Galápagos, Ecuador; Department of Biological Sciences, Northern Illinois University, De Kalb, IL, USA; Department of Biological Sciences, Kent State University, Kent, OH, USA; School of Biomedical Sciences, Kent State University, Kent, OH 44240, USA

**Keywords:** avian vampire fly, Galápagos, population genomics

## Abstract

Invasive species pose significant threats to island ecosystems, often leading to the decline of native species and the disruption of ecological balance. The avian vampire fly (*Philornis downsi*), introduced to the Galápagos Islands of Ecuador, has emerged as a major threat to the endemic avifauna, parasitizing multiple species of Darwin's finches and other passerines. Yet, the genetic mechanisms of its invasion remain unclear. In this study, we conducted the first whole-genome sequencing analysis of *P. downsi* populations from the Galápagos Islands and their native range in mainland Ecuador. Our results reveal genomic signatures of a founder effect, with reduced genetic diversity in the Galápagos populations, indicative of a recent population bottleneck. We found a lack of significant genetic differentiation and evidence of ongoing gene flow among island populations. Despite low genetic diversity in island populations, we identified adaptive genetic changes, including regions possibly under positive selection near genes related to neural signaling, muscle development, and metabolic processes, which may have contributed to the fly's invasion success. Additionally, we uncovered genetic changes associated with precipitation-related climate adaptation, highlighting the possible role of environmental factors in shaping genetic variation in *P. downsi*. Our findings provide crucial insights into the invasion dynamics of *P. downsi* in Galápagos, emphasizing the importance of genomic research in informing conservation strategies. The identification of key adaptive genomic loci and potential environmental drivers of genetic change will aid in the development of targeted management practices to mitigate the impact of this invasive species on the unique biodiversity of the Galápagos Islands.

## Introduction

Increasing globalization has significantly altered species’ movement patterns across geographical borders. This enhanced dispersal has led to the introduction of diverse organisms into environments beyond their native habitats and resulted in the establishment of self-sustaining populations in new areas. In the absence of natural predators or competitors that keep them in check in their native habitats, some of these introduced alien species outcompete local biodiversity (Pilliod et al. 2012). This enables them to rapidly proliferate, disrupt the ecological balance, and pose threats to ecosystem stability qualifying them as invasive species (Sladonja et al. 2018). Through these changes, invasive species can change the ecological and evolutionary trajectories of native species, in many cases driving them to extinction (Mooney and Cleland 2001). Among introduced alien species, insects constitute a significant category, with over 7,000 species currently established beyond their native habitats and some of these causing considerable harm (Seebens et al. 2017; Gippet et al. 2019; Bonnamour et al. 2023). Projections suggest a 36% increase in the number of insect invasions from 2005 to 2050, underscoring the escalating impact of insect invasions on global ecosystems and highlighting the need for comprehensive strategies to address and mitigate the consequences of these invasions (Hui and Richardson 2017; Abram et al. 2024).

The impact of invasive species can be particularly severe in fragile ecosystems like islands, which often harbor endemic species with small population size (Spatz et al. 2017; Burgess et al. 2021). For example, many species of endemic honeycreepers have gone extinct or are critically endangered primarily due to an invasive avian malaria parasite (*Plasmodium relictum*) that is vectored by an invasive mosquito (*Culex quinquefasciatus*) in the Hawaiian archipelago (Villena et al. 2024). Identifying the invasion routes and the biological pathways that contribute to the success of non-native invasive species is critical for understanding the dynamics of invasions and customizing effective management strategies. Genomic resources have emerged in recent times as invaluable tools for bridging gaps in our understanding of invasion pathways (McCartney et al. 2019). Genomic tools can be used to identify source populations, determine the number and size of their introductions (Jaspers et al. 2021), reconstruct invasion routes (North et al. 2021), and monitor invasive populations by characterizing their demographic and evolutionary history (McCartney et al. 2019; Chen et al. 2024). A key emphasis in the studies of invasive species has been on identifying and characterizing the traits that contribute to their success (Parker et al. 2013). However, there is still a significant gap in our understanding of the genetic mechanisms that underlie these invasive traits (Matheson and McGaughran 2022). One such example is the invasion dynamics of the avian vampire fly (*Philornis downsi*) in the Galápagos Islands of Ecuador.

*Philornis downsi* is a Neotropical muscid fly native to mainland South America and the island of Trinidad (Dudaniec and Kleindorfer 2006; McNew and Clayton 2018; Bulgarella et al. 2019; Common et al. 2020). It was introduced to the Galápagos Islands, where it has persisted since at least the 1960s and has become a significant invasive species on a majority of the islands within the archipelago (Fessl et al. 2018). In its larval stage, the fly parasitizes most of the passerine species in Galápagos, including at least 11 species of Darwin's finches (Fessl et al. 2018; Coloma et al. 2020; Anchundia and Fessl 2021), feeding on the blood and other fluids of nestlings and brooding adult birds as an obligate nest ectoparasite (Fessl and Tebbich 2002). The invasion of *P. downsi* has already led to population declines in two species of Darwin's finches listed by the IUCN as critically endangered; the Medium Tree Finch (*Camarhynchus pauper*) and the Mangrove Finch (*Camarhynchus heliobates*) (Dvorak et al. 2004; Grant et al. 2005; Cimadom et al. 2014; Peters and Kleindorfer 2018; Bulgarella et al. 2019) The fly is now considered one of the greatest threats to the unique and endemic bird species of the Galápagos Islands (Causton et al. 2013; McNew and Clayton 2018).

Previous population genetic studies of *P. downsi* in Galápagos have used reduced sets of molecular markers such as mitochondrial DNA, microsatellites, and single-digest restriction site-associated DNA sequencing (RADseq) (Dudaniec et al. 2008; Koop et al. 2021). These studies have revealed a high degree of relatedness and a lack of genetic differentiation among island populations, suggesting the possibility of ongoing gene flow. However, a comprehensive genome-wide study of *P. downsi* was still lacking. In this study, we used a whole-genome sequencing approach to conduct population genomics analyses of *P. downsi* populations from the Galápagos Islands and its native range, mainland Ecuador. By examining genetic diversity, population structure, and adaptive genetic potential, we aimed to understand the mechanisms underlying the fly's successful invasion in the Galápagos Islands. Our study presents the first whole-genome characterization of *P. downsi* populations and contributes insights to help aid the monitoring and management efforts of this species.

## Results and Discussion

### Reduced Genetic Diversity and Signatures of the Founder Effect in *P. downsi* Populations in the Galápagos Islands

**Fig. 1.**
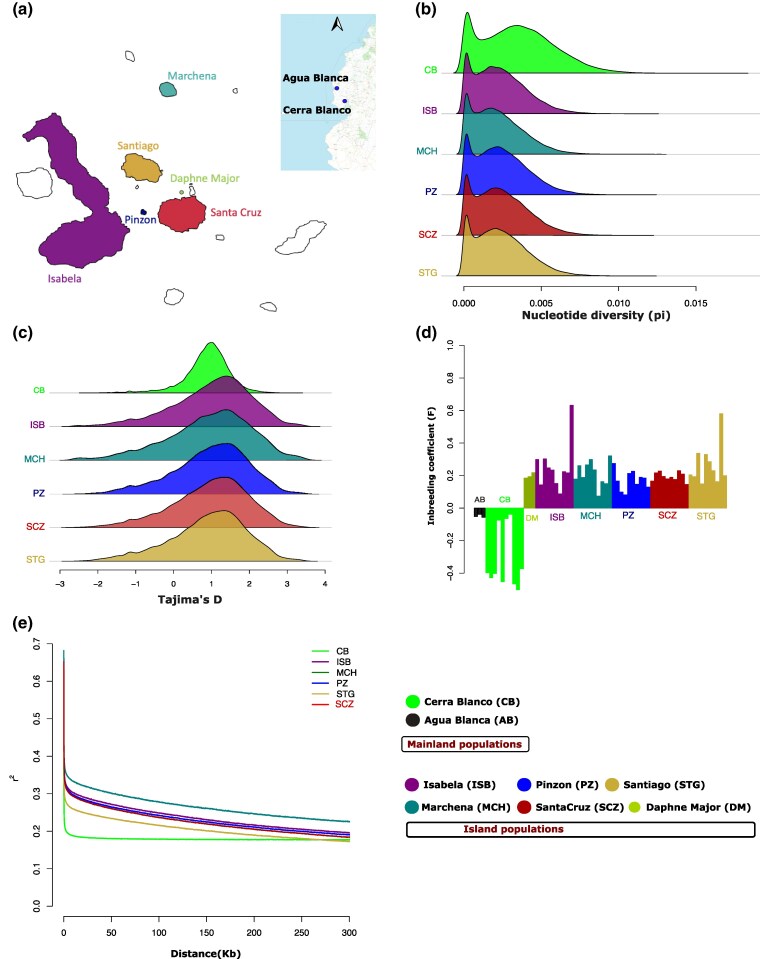
Genomic signatures of founder effect in *P. downsi* populations in the Galápagos Islands. a) Sampling locations. We collected samples from two mainland populations and six island populations from Galápagos. The mainland samples are shown as inset. The maps of the mainland and Galápagos are not in the same scale. b) Distribution of average nucleotide diversity (pi) in 15-kb windows across the genome. c) Distribution of average Tajima’s D in 15-kb windows across the genome. Samples from AB and DM are excluded from b) and c), to minimize sample size bias. d) Inbreeding coefficient score (F) for each individual. e) The decay of pairwise linkage disequilibrium (LD) score between SNPs up to a distance of 300 kb.

We carried out population-scale whole-genome sequencing of 53 individuals of *P. downsi* from six islands in Galápagos, and 13 individuals from two different locations in mainland Ecuador (Fig. 1a, supplementary table S1, Supplementary Material online). Each individual was sequenced to ∼20× coverage, and reads were aligned to the previously published *P. downsi* genome assembly (Romine et al. 2022) to identify ∼12.4 million single nucleotide polymorphisms (SNPs). We characterized the genetic diversity of *P. downsi* populations from mainland Ecuador and the Galápagos Islands using several population genetic statistics: nucleotide diversity, Tajima's D, inbreeding coefficient, and linkage disequilibrium (LD). The results from each independent analysis consistently indicated that the genetic diversity in Galápagos populations of *P. downsi* is lower compared to mainland populations.

The genome-wide average nucleotide diversity (pi) in island populations was lower than in the mainland populations included in this study (Fig. 1b, supplementary table S2, Supplementary Material online), indicating the reduced genetic diversity in island populations. Similarly, the genome-wide average Tajima's D was higher in island populations compared to the mainland (Fig. 1c, supplementary table S3, Supplementary Material online), indicating fewer rare alleles in the island gene pool, suggesting a recent bottleneck event likely as a consequence of the founder effect due to the invasion and colonization of *P. downsi* in the Galápagos islands. We further calculated the inbreeding coefficient (F) for each individual as a measure of average genome-wide homozygosity for each sample. F scores for mainland samples were close to zero, whereas island samples had higher positive scores (Fig. 1d), indicating higher homozygosity and reduced genetic variation in the island populations. We also estimated pairwise linkage disequilibrium (LD) between all polymorphic SNPs in each population. We identified that LD decays slower in the island populations, compared to the mainland population (Fig. 1e), consistent with a founder effect, where a small initial population size leads to nonrandom mating and reduced recombination (Provine 2004).

The findings from these population genetic analyses collectively suggest a founder effect in *P. downsi* populations in Galápagos. The concept of a founder effect, where a small number of individuals establishes a new population, is well-documented in invasive species (Nei et al. 1975). This small founding population carries only a fraction of the genetic variation present in the source population, resulting in reduced genetic diversity even after the population has expanded. Previous population genetic studies on *P. downsi* in Galápagos using mitochondrial, microsatellite, and RADSeq data (Dudaniec et al. 2008; Koop et al. 2021), identified low levels of genetic differentiation between *P. downsi* populations on the Galápagos islands. The results based on our high-resolution whole-genome data are consistent with these previous findings. Interestingly, we show here that an invasive parasite is subject to the same evolutionary pitfalls (i.e. reduced genetic variation because of founder effects and genetic bottleneck) as are nonparasitic species (Mittan-Moreau et al. 2022). Given that *P. downsi* have a direct parasitic life cycle and likely invaded independently of their host species (Fessl et al. 2018), this result is perhaps to be expected. Thus, while not our primary goal, our results add to a small but growing body of literature investigating whether parasites show differential signatures of colonization than nonparasitic species (Koffi et al. 2006; Blakeslee et al. 2020; Sromek et al. 2023).

### Lack of Genetic Differentiation Among Islands, Population Connectivity, and Gene Flow in *P. downsi* in Galápagos

**Fig. 2.**
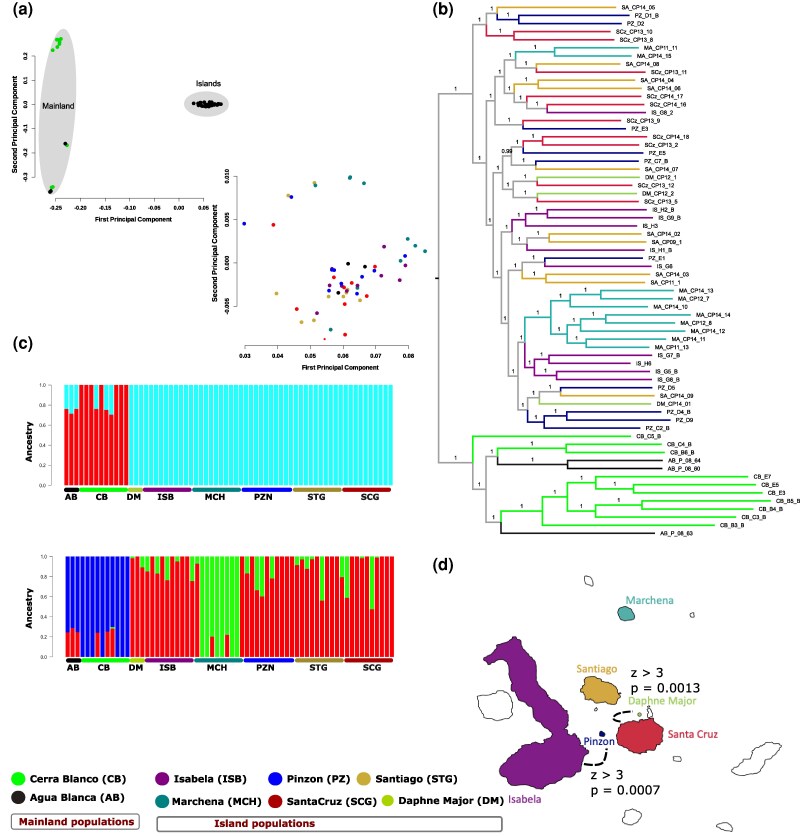
No genetic differentiation among island populations and evidence of gene flow. a) Principal component analysis (PCA) indicates clear separation among mainland and island populations. A tight clustering of all island samples compared to the mainland indicated a significantly low amount of genetic variation in island populations. Mainland samples, however, appear spread across the two main principal components in the PCA, showing that they are much more genetically diverse than the island populations. PCA plot showing only island populations (right panel) is based on separate analysis. b) Maximum likelihood phylogenetic tree generated using 12,442,422 biallelic SNPs and 66 individuals shows two main groups: the mainland and the islands. Among the islands, the individuals did not cluster separately into separate groups, except for the majority of individuals from Marchena Island (eight out of ten) which grouped into a separate clade. c) Admixture analyses show K = 2 and 3 as the more likely population structure for all 66 samples. In K = 2 (upper panel), the individuals are grouped into their mainland and island populations. In K = 3 (lower panel), the mainland samples form a separate group, eight samples from Marchena (out of ten) form the second group, and the third group is the samples from all the other islands used in this study. d) Evidence of gene flow among populations from Santa Cruz, Daphne Major, Isabela, and Pinzón (*P* < 0.001 and Z > 3) and their respective location in the Galápagos archipelago.

To examine the genetic relationship among mainland and island populations of *P. downsi*, we performed principal component analysis (PCA) using LD-pruned SNP data (as described in Materials and Methods). The main components of genetic variation (PC1 and PC2) separated the two mainland populations from the six island populations (Fig. 2a). A tight clustering of all island samples compared to the mainland indicated a significantly low amount of genetic variation in island populations, which is consistent with other results of genetic signatures of founder effect discussed above. We further performed PCA using island populations only (Fig. 2a), which indicated that most individuals from the islands did not cluster separately into genetically distinct groups. However, individuals from Marchena Island appeared relatively genetically distinct compared to the individuals from the other islands. Marchena is the most isolated of the sampled islands, approximately 54 km to the North of the next closest island, Santiago. Mainland samples, however, appear spread across the two main principal components in the PCA, showing that they are much more genetically diverse than the island samples. This indicates significant genetic divergence of island populations from the mainland but relative genetic homogeneity among the islands.

We used the SNP data to build a phylogenetic tree using a maximum-likelihood approach (Price et al. 2010), which highlighted a clear separation between mainland and island populations, with island populations forming a distinct clade (Fig. 2b). This supports the PCA findings of significant divergence of island populations from the mainland. Among the islands, the individuals did not cluster into separate groups, except for the majority of individuals from Marchena Island (eight out of ten) which grouped into a single clade, indicating close genetic relationships and low differentiation among the majority of island populations. Our PCA analysis and phylogenetic tree also indicated that two mainland populations were not genetically distinct, as three out of ten individuals from Cerra Blanco grouped with individuals from Agua Blanca (Fig. 2a/2b). The straight-line distance between the Agua Blanca and Cerro Blanco is 113 km, and both are located within the Chongon–Colonche mountain range, which stretches from coastal Manabi Province southeast to the city of Guayaquil. Thus, some degree of gene sharing between these mainland populations is to be expected. However, additional sampling of mainland populations will be required for better characterization of the genetic diversity of *P. downsi* across its native range in mainland Ecuador.

We also conducted population ancestry analysis using ADMIXTURE (Alexander et al. 2009) and assessed various values of K using a cross-validation procedure. We identified that k = 2 or 3 is optimal for admixture analysis (supplementary table S4, Supplementary Material online). For k = 2, mainland and island populations were separated, and for k = 3, we found that the Marchena population was genetically distinct from the rest of the island populations (Fig. 2c). Population ancestry analysis revealed that Galápagos populations predominantly share a common ancestry distinct from mainland populations. These results highlighted that there was minimal genetic input from the mainland, indicating that either (i) there is a strong founder effect and subsequent isolated evolution, and/or (ii) that the mainland populations sampled were not the primary source populations for the invasion into Galápagos.

Our finding that the populations of *P. downsi* from different Galápagos islands are not genetically distinct, could be attributed to either a relatively recent invasion with insufficient time for genetic divergence or to the possibility of ongoing gene flow among the islands. These two processes are not mutually exclusive, and both could be contributing to the observed genetic homogeneity across island populations. We tested for evidence of gene flow between island populations by calculating Patterson's D (ABBA-BABA) statistics implemented in Dsuite (Malinsky et al. 2021). We identified evidence in support of ongoing gene flow between the islands that are geographically closer (i) between populations of Santa Cruz and Daphne Major and (ii) Isabela and Pinzón (D > 3, *P* < 0.01) (supplementary table S5, Supplementary Material online, Fig. 2d), suggesting that these populations are not isolated and there is more movement of individuals between these islands than those further away from one another. Conversely, we found no evidence of gene flow associated with the Marchena population, suggesting that this population is genetically isolated from the others.

The natural dispersal mechanisms of *P. downsi* could contribute to gene flow among island populations. Adult flies are strong flyers and have the potential to move between islands on their own (Fessl et al. 2018), possibly facilitating genetic exchange. They are also long-lived in the field (Bulgarella et al. 2022) which could increase the chances of inter-island dispersal. Lomas (2008) previously found five adult *P. downsi* onboard a tourist boat that was traveling between islands, while in a more recent study by Causton et al. (unpublished data), a gravid female was trapped onboard a tourist boat. That said, further research is needed to confirm the dispersal distances and patterns of *P. downsi* as we do not have sufficient information on their natural movement (Fessl et al. 2018). However, our results indicate evidence of gene flow among islands that have human settlements and/or are popular tourist destinations in Galápagos, such as Santa Cruz and Isabela, and islands such as Daphne Major and Pinzón that are close to these islands. As these islands experience increased human activity, movement of goods, and tourism-related travel, we hypothesize that human activity is a key factor that has facilitated the gene flow among *P. downsi* populations in Galápagos. The absence of gene flow associated with the less visited and more isolated Marchena island (there are no tourist visitor sites on this island), indicates that isolation by distance (regardless of dispersal mechanism) likely plays a strong role in shaping the population genetic structure of *P. downsi* in Galápagos.

Another hypothesis that could explain the genetic differentiation of the Marchena population is the possibility of a second invasion event, where the founding population on this island may have originated from a different mainland source population than those that colonized the other islands. However, our results indicate that individuals from Marchena are genetically more similar to other island populations than to the mainland populations analyzed in this study. When founding individuals originate from multiple source populations, the genetic diversity of the founder population can be relatively high (Dlugosch and Parker 2008). The genetic diversity in the Marchena population is not higher than that of other island populations and the source population of the invasion is still unknown. To thoroughly test the hypothesis of single or recurrent invasion events, future studies should aim to characterize additional populations of *P. downsi* in its native range on the mainland. This could provide a broader understanding of the genetic structure and invasion history of *P. downsi* in the Galápagos Islands.

### Adaptive Potential of *P. downsi* and Genetic Mechanisms of Its Successful Invasion in Galápagos

Reduced genetic diversity in a population typically indicates reduced adaptive potential (Boulding 2008). However, the successful invasion of *P. downs*i in Galápagos, despite lower genetic diversity, raises intriguing questions about the underlying genetic mechanisms enabling this adaptation. Previous studies have highlighted the ecological flexibility of *P. downsi*, emphasizing factors such as reduced host specificity, a broad host range, the absence of natural enemies, adaptability to a wide range of habitat types, a high dispersal ability, and adult longevity, all of which could have contributed to their successful invasion (Causton et al. 2006, 2013, 2019; Bulgarella et al. 2015, 2017, 2022; Fessl et al. 2018; McNew and Clayton 2018; Common et al. 2020).

**Fig. 3.**
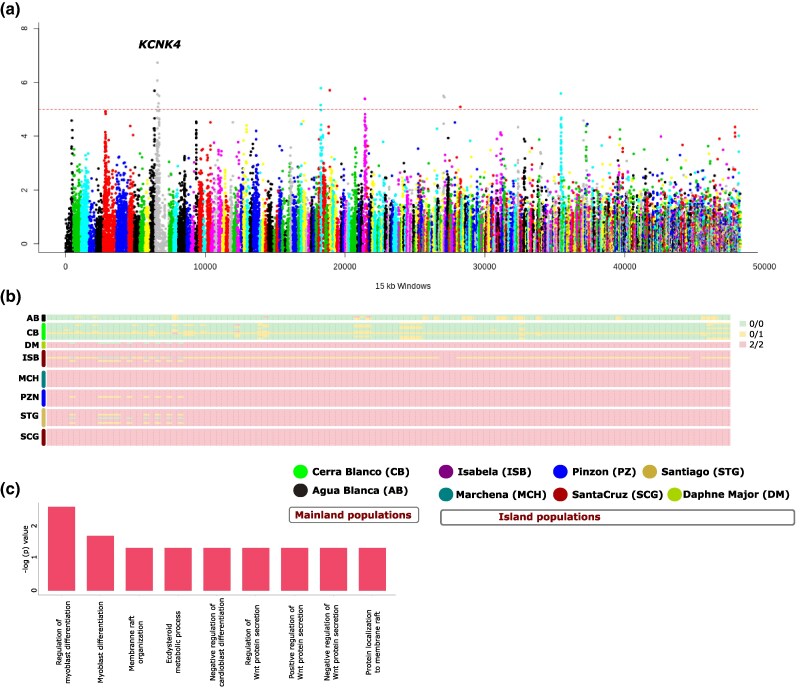
Genomic signals of positive selection and local adaptation in island populations. a) Distribution of ZF_ST_ score in 15-kb windows across the genome between the mainland (n = 13) and island (n = 53) populations. The *KCNK4* gene overlapping the genomic region showing the strongest divergence between mainland and island populations is indicated. b) Genotype pattern in 184 SNPs within the genomic region showing the strongest divergence between mainland and island populations. Mainland and island populations appear to be fixed for two different haplotypes at this locus. c) Significantly enriched Gene Ontology (GO) categories (*P* < 0.01) among 117 genes within 100-kb upstream and downstream of the genomic regions showing strong divergence (ZF_ST_ > 5) between mainland and island populations.

To uncover the genetic mechanisms enabling the successful invasion of *P. downsi* in Galápagos, we screened the mainland and island populations to identify genomic regions with the highest fixation indices (F_ST_), which is indicative of loci under strong positive selection. The F_ST_ distribution was Z-transformed (ZF_ST_) and sixteen 15-kb windows from eight scaffolds with top ZF_ST_ values (ZF_ST_ > 5) (Fig. 3a) were selected as candidate genomic regions showing strong genetic divergence between mainland and island populations of *P. downsi*. The region showing the strongest genetic divergence between mainland and island populations was a 15-kb window (scaffold 54: 3,270,001 to 3,285,000) with a ZF_ST_ of 6.73 (supplementary table S6, Supplementary Material online). One hundred twenty out of 184 SNPs in this window (65.22%) were close to fixation (F_ST_ > 0.8), with mainland and island populations appearing to carry two distinct haplotypes (Fig. 3b). Only one individual from Cerra Blanco (mainland) and Isabela (island) were heterozygous at this locus. This divergence suggests that the genomic region is close to fixation, indicative of a selective sweep. The presence of distinct haplotypes between mainland and island populations suggests that the alleles within this region confer specific adaptive advantages that have been favored in the Galápagos environment.

We further investigated the functional roles of genes located near regions with high fixation indices (F_ST_) (supplementary table S6, Supplementary Material online). To determine whether these high-F_ST_ regions overlap with regulatory elements linked to nearby genes, we extracted 117 genes within 100-kb upstream and downstream of these genomic regions (supplementary Data S1, Supplementary Material online) and analyzed their functions and ontology. While regulatory regions can span from a few hundred base pairs (e.g. proximal promoters) to several hundred kilobases (e.g. distal enhancers), a 100-kb window provides a reasonable balance for identifying possible proximal and distal regulatory elements, as used in previous studies in other species (Li et al. 2022; Wang et al. 2022; Crespo-Piazuelo et al. 2023). Enrichment analysis revealed that the majority of significantly enriched GO terms (adjusted *q* < 0.05) were involved in the regulation of (i) myoblast differentiation, (ii) Wnt protein secretion, (iii) membrane rafts, and (iv) ecdysteroid metabolic process (Fig. 3c). We also examined the enrichment of KEGG metabolic pathways (Kanehisa et al. 2016), and this list of genes was significantly enriched (adjusted *q* < 0.05) for the Pentose phosphate pathway.

These enriched gene functions collectively provide a foundation for formulating hypotheses to understand the physiological and developmental adaptations of *P. downsi* in Galápagos. For example, Potassium channel genes such as *KCNK4* (gene overlapping the region showing strongest genetic divergence) and those involved in myoblast differentiation regulate muscle contractions (Jackson 2017) and play key roles in muscle development (Tomaz da Silva et al. 2023). Selective divergence on these genes possibly confers a fitness advantage for *P. downsi* for enhancing their flight capability which could facilitate dispersal within and between islands, allowing them to exploit new habitats and resources. The enrichment of other genes involved in cell signaling, membrane organization, and hormone regulation, possibly underscores the multifaceted nature of *P. downsi*'s adaptation in Galápagos. While our findings provide insights into developing a hypothesis on potential genetic mechanisms underlying the invasion success of *P. downsi* in Galápagos, they also come with possible limitations, particularly concerning the challenge of disentangling drift from selection in shaping the observed genomic patterns. Future studies on experimental validation (such as functional assays), or associations between these genes and fitness-related traits, would be necessary to confirm their adaptive significance.

### Genetic Changes Associated With Possible Climate Adaptation in *P. downsi*

**Fig. 4.**
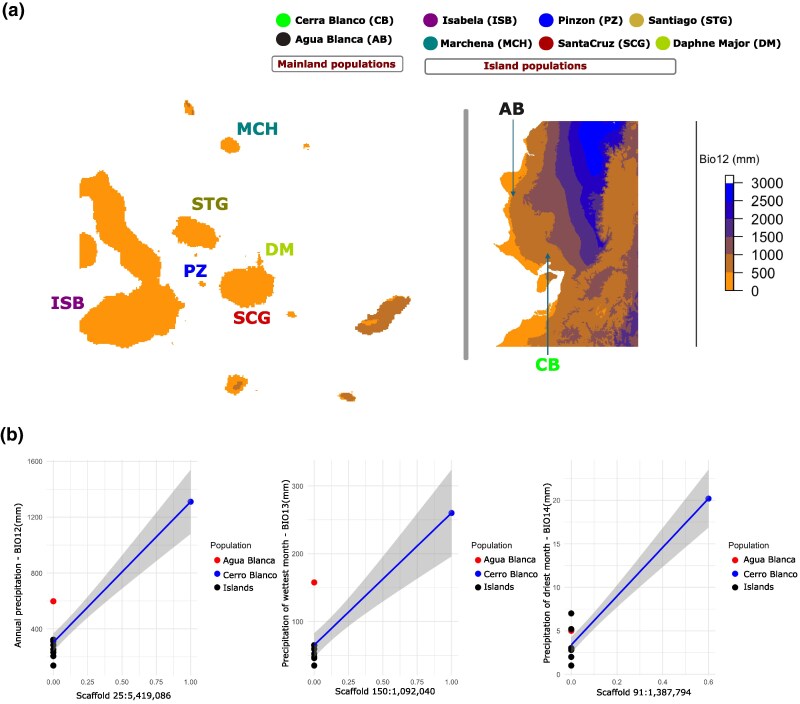
Inference of gene–environment associations. a) Average annual precipitation (mm) in the Galápagos Islands and mainland Ecuador. Different scales are used for plotting mainland and islands to highlight variation. The maps of the mainland and Galápagos are not in the same scale. b) Correlation of population allele frequency of top candidate SNPs that are significantly associated with three precipitation-related bioclimatic variables; left panel: annual precipitation (BIO12), middle panel: precipitation of wettest month (BIO13), and right panel: precipitation of driest month (BIO14).

We further examined whether genetic changes between mainland and island populations or within island populations are associated with climatic variables. We extracted 19 bioclimatic variables from the WorldClim database (Fick and Hijmans 2017) associated with temperature and precipitation measurements from the mainland (Ecuador) and the Galápagos islands locations used in this study. Three environmental predictors related to precipitation (BIO12 = annual precipitation, BIO13 = precipitation of wettest month, and BIO14 = precipitation of driest month) showed the greatest variation among the study sites (Fig. 4a, supplementary table S7, Supplementary Material online). For example, the average precipitation of the wettest month was 1,310.4 mm for Cerro Blanco (mainland) while it was 137 mm for the island Daphne Major. However, it is important to note that this data set may have significant gaps, as climate variables for many islands are poorly sampled. For instance, the higher rainfall experienced in the highlands of some Galápagos islands is not adequately reflected in the available data.

To investigate genomic loci potentially involved in climate adaptation associated with precipitation, we used the latent factor mixed model (LFMM), a univariate analysis that considers population structure as a latent factor and finds an association between each SNP and the specific environmental variable (Frichot et al. 2013). We identified 555 SNPs that were significantly associated with mean annual precipitation (BIO12), 361 SNPs associated with precipitation of the wettest month (BIO13), and 1,596 SNPs associated with precipitation of the driest month (BIO14) (supplementary fig. S1, Supplementary Material online). The correlation between environmental variables and allele frequency exhibited a similar profile for all top candidate SNPs (Fig. 4b, supplementary fig. S2, Supplementary Material online). The mainland population from Cerro Blanco which received the highest precipitation, was fixed for one allele, whereas the other mainland population from Agua Blanca whose precipitation levels were lower and similar to those in the Galápagos islands, was fixed for the other allele. The fact that these two mainland populations are fixed for different alleles, with one population (Agua Blanca) sharing allele frequencies similar to those of the island populations, suggests that the genetic variation observed could be linked to climate adaptation, particularly precipitation. However, we also acknowledge the need for further studies to confirm whether these alleles are directly related to precipitation-related selection or if other factors, such as population history may have contributed to the observed allele distributions.

Among the genes overlapping the candidate SNPs that are significantly associated with each of the three bioclimatic variables (BIO12, BIO13, and BIO14), there were 24 genes shared among all three sets (supplementary Data S2, Supplementary Material online). We define these genes as “core” candidate genomic loci that are possibly associated with precipitation-related climate variables. The majority of these genes were annotated as uncharacterized proteins, limiting the ability to perform enrichment analysis of Gene Ontology (GO) terms and KEGG metabolic pathways. However, we identified one gene (*MTMR2*), which codes for myotubularin-related protein 2, which is linked to drought response in Arabidopsis (Ding et al. 2009), suggesting potential mechanisms for adaptation to varying water availability. The Galápagos Islands have drastic variations in climate conditions within and between years, with regular El Nio and La Niña events (Grant 2014). These genetic changes may have improved the flies’ ability to retain water and maintain metabolic functions during the changing seasons in Galápagos (Trueman and d’Ozouville 2010). However, future experimental studies are needed to validate these hypotheses.

### Implications of This Genomic Research for the Conservation and Management of *P. downsi* in the Galápagos Islands

The findings from this study have several implications for the conservation and management of *P. downsi* in the Galápagos Islands. Currently, several management strategies are being employed or being considered to control fly populations, including insecticide applications, mass-trapping, and the release of biocontrol agents (Knutie et al. 2014; Cha et al. 2016; Fessl et al. 2018; Yuval et al. 2019; Bulgarella et al. 2020; Boulton et al. 2024). Our studies have revealed low genetic differentiation among populations of *P. downsi* across the Galápagos islands, coupled with evidence of gene flow, showing that populations from different islands are more interconnected than previously thought. Management strategies should therefore consider the interconnectedness of populations such that control measures on one island might need to be complemented by efforts on neighboring islands to effectively reduce gene flow and manage re-colonization. Resources can be targeted more efficiently by focusing on islands with high levels of gene flow, such as those frequented by tourists, to prevent further spread.

In mainland Ecuador, the parasitic wasp, *Conura annulifera*, parasitizes *P. downsi* pupae (Bulgarella et al. 2017; Ramirez et al. 2022). The wasp species, parasitoids of *Philornis*, are exceedingly rare in Galápagos and only include generalist species (Lincango and Causton 2008; Fessl et al. 2018, Bulgarella et al. 2022). Thus, the introduction of specialized parasitoid wasps from the native range of *P. downsi* (biological control) that impose minimal risk to native Galápagos fauna is a promising strategy to manage or control *P. downsi* (Bulgarella et al. 2017; Fessl et al. 2018; Boulton et al. 2019; Ramirez et al. 2022). Our study begins to explore the comparative genetic variability of fly populations found on the mainland and the Galápagos Islands. The reduced genetic variability of island populations may increase their susceptibility to these wasps, though, to our knowledge, no studies have yet looked for genotype-associated virulence metrics with regard to these species. Furthermore, our study provides a framework for which future studies can consider the selective consequences of this management strategy.

This study highlights potential genetic mechanisms that may have facilitated the successful adaptation of *P. downsi* to the Galápagos environment. The identification of genes possibly associated with successful adaptation offers valuable targets for further research. Future functional genomic studies, such as gene expression analyses, CRISPR-based functional assays, and transcriptomic profiling, could provide deeper insights into the precise roles these genes play in adaptation. These studies will help uncover the underlying molecular mechanisms and validate the functional significance of the identified candidate genes. Ultimately, such research could inform more precise management strategies that address specific adaptations and vulnerabilities, improving efforts to manage the species and mitigate its impact on the Galápagos ecosystem.

## Materials and Methods

### Sampling

We reared adult individuals from *P. downsi* pupae collected from nests of different species of birds in two localities of mainland Ecuador (three individuals from the Agua Blanca section of Machalilla National Park, Manabi Province, and ten individuals from Cerro Blanco Protected Forest, in Guayaquil, Guayas Province). All Agua Blanca samples came from a single wild Streaked Flycatcher (*Myiodynastes maculatus*) nest containing 28 *P. downsi* puparia, and all Cerro Blanco samples came from a single nest of a Sooty-crowned Flycatcher (*Myiarchus phaeocephalus*), that had been constructed in an artificial nest box (as in Bulgarella et al. 2015) and contained 32 *P. downsi* puparia. *Philornis downsi* developing in the same host nest are not necessarily siblings since multiple *P. downsi* females are known to contribute eggs to bird nests (Dudaniec et al. 2010; O’Connor et al. 2010; Pike et al. 2021). Similarly, we collected 53 individuals from six islands in the Galápagos archipelago; Isabela (Sierra Negra Volcano), Daphne Major, Marchena, Pinzón, Santa Cruz, and Santiago (supplementary table S1, Supplementary Material online) using either adults that emerged from pupae collected from bird nests (mainland samples; Bulgarella et al. 2015) or adults captured in traps baited with fermenting papaya juice (Galápagos samples; Lahuatte et al. 2016). Collections were done between 2015 and 2022. The samples were collected in ethanol (96%) and stored at −80 °C before DNA extraction.

### Whole-Genome Sequencing, Alignment, and Variant Calling

We extracted total DNA from the abdomen of the flies using the Qiagen DNeasy blood and tissue kit (https://www.qiagen.com/us, Cat. No. 69504) following the animal tissue extraction protocol with an overnight digestion step. DNA extractions were quantified using a Qubit 4 fluorometer (Invitrogen, https://www.thermofisher.com/). Whole-genome libraries with an average fragment size of 350 bp were prepared and sequenced using an Illumina NovaSeq platform to produce 150 bp paired-end reads aiming to produce 15 Gb of raw data per individual. Libraries were prepared and sequenced by Novogene (https://www.novogene.com/) and read quality was checked using FASTQC (https://www.bioinformatics.babraham.ac.uk/); all read files from all individuals passed the quality check.

We mapped the cleaned reads against the reference genome of the avian vampire fly, we had generated before (Romine et al. 2022) using BWA with default parameters (Li and Durbin 2009). SAM files obtained from BWA were converted to BAM files, and sorted using SAMtools (Danecek et al. 2021). The sorted alignments were then checked for PCR duplicates using Picard (https://broadinstitute.github.io/picard/). Variant calling per individual was done using the HaplotypeCaller tool from the Genome Analysis Toolkit GATK (McKenna et al. 2010; Poplin et al. 2018). We then used GATK tools CombineGVCFs and GenotypeGVCFs to combine the variants per individual into a single VCF file.

We examined the variant quality by checking the distribution of the VCF statistics (mean depth, and sequence quality score), and by checking missing genotype data per individual using VCFtools (Danecek et al. 2011, 2021). We filtered the VCF file to select variants only genotyped in all individuals (no missing data), only biallelic SNPs (excluding indels and variants with more than two alleles), mean depth (DP) between 8 and 25, and minimum allele frequency of 0.02. After filtering we kept a total of 12,442,422 biallelic SNPs that were variable within or between populations, which were used for downstream analyses.

### Population Genetics Statistics

We used VCFtools (Danecek et al. 2011) to calculate two measures of population genetic diversity (nucleotide diversity and Tajima's D). To address sample size bias in such estimates, we only used populations with equal sample size (n = 10) in the analysis. We also calculated the inbreeding coefficient (F) as a measure of average genome-wide homozygosity for each sample using a method of moments implemented in VCFtools. We further used ngsLD (Fox et al. 2019) to estimate pairwise LD that takes the uncertainty of genotype's assignation into account, using the whole-genome SNP data in each *P. downsi* population.

### Population Genetic Structure and Ancestry

We examined the genetic differentiation among samples using a PCA with Plink v.1.9 (Chang et al. 2015). As one major assumption of a PCA is independent data, we first pruned our SNP data set considering LD. We removed any SNP that showed an *r*^2^ > 0.1 within a 50-kb window and step size of 10 bp for conducting the PCA. We plotted the PCA output in R (Team 2016). We further estimated the ancestry of individuals based on a genome-wide SNP data set using Admixture (Alexander et al. 2009). We ran the population ancestry analysis using K = 1 to 10. To determine the optimal number of genetically distinct clusters (K) that best represent our data, we conducted an exploratory analysis using a cross-validation (CV) procedure. This analysis was performed with the K-means method implemented in Admixture. We compared the CV error values of the different K runs to choose the more likely number of genetic clusters in our data.

### Phylogeny Reconstruction and Population Structure

For phylogeny reconstructions, we produced concatenated fasta files excluding ambiguous characters (IUPAC characters for heterozygous positions) using vcf2phylip (Ortiz 2019). We used the fasta files to build maximum likelihood phylogenetic tree using the GTR gamma model in FastTree (Price et al. 2010). For visualizing the trees, we used FigTree v1.4.4 (https://github.com/rambaut/figtree). Local support values for each node were estimated using Shimodaira–Hasegawa test implemented in FastTree.

### Examination of the Evidence of Gene Flow

Using our filtered SNP data set of ∼12 million SNPs, we employed ABBA-BABA tests, a statistical technique designed to detect and measure gene flow between populations by analyzing allele-sharing patterns at particular genomic loci, utilizing Dsuite (Malinsky et al. 2021). We examined the possible evidence of gene flow between all possible combinations of *P. downsi* populations.

### Genomic Signatures of Selection Associated With Invasive Success in the Galápagos Islands

We scanned the genome in nonoverlapping 15-kb windows to estimate pairwise F_ST_ values between mainland and island populations using VCFtools (Danecek et al. 2011). We further normalized the mean F_ST_ score in each 15-kb genomic window using Z normalization using custom codes in R (https://www.R-project.org/). We selected the genomic windows with ZF_ST_ > 5 as candidate regions for downstream gene function and enrichment analysis.

### Inference of Gene–Environment Associations

We obtained 19 bioclimatic variables for the period 1970 to 2000 with a spatial resolution of 30 s (∼1 km²) from the WorldClim database, covering mainland Ecuador and the Galápagos Islands (Fick and Hijmans 2017). For our analysis, we focused on three precipitation-related variables: BIO12 (annual precipitation), BIO13 (precipitation of the wettest month), and BIO14 (precipitation of the driest month). We prioritized these precipitation variables because the differences in precipitation patterns between the mainland and the islands were the most pronounced for these variables. For instance, the average BIO13 value for Cerro Blanco was 1,310.4 mm, compared to just 137 mm on Daphne Major Island.

To perform this analysis only on SNPs that were shared among populations, we selected SNPs with minor allele frequency > 0.05 and used the filtered SNP data to run LFMM, a univariate analysis that considers population structure as a latent factor and finds associations between each SNP and respective environmental factor (Frichot et al. 2013). Our objective was to find loci that exhibit significant deviations from the general genetic structure of the population and have a strong association with precipitation. We used three latent factors in LFMM based on the number of ancestry clusters inferred in our population ancestry analysis using Admixture. To account for the population structure in the genotype data, five independent Markov Chain Monte Carlo runs were conducted for each environmental variable, with 5,000 iterations used as burn-in and 10,000 iterations, as suggested (Frichot and François 2015). The obtained *P*-values were Z-normalized and adjusted for the genomic inflation factor. A false discovery rate correction of 3% was applied to adjust for multiple testing errors.

### Functional Annotation of Candidate Genomic Regions

Functional annotation of 53,760 transcripts previously identified in the *P. downsi* genome (Romine et al. 2022) was performed using the eggNOG-mapper pipeline (version 2) (Cantalapiedra et al. 2021) with the default parameters for characterizing GO terms and KEGG pathways. Based on these results, a specific organism database (OrgDB) library for *P. downsi* was constructed using the makeOrgPackage function of the R package AnnotationForge (Carlson and Pagès 2019). Additionally, the mapping between KEGG pathway IDs, KEGG pathway names, and gene IDs was built using a custom-built R script. These results were subsequently utilized to perform GO enrichment analysis and KEGG pathway enrichment analysis using the enrichGO and enricher functions from the R package clusterProfiler (Wu et al. 2021). Both adjusted *P*-values and *q*-values less than 0.05 were considered statistically significant for enrichment.

## Supplementary Material

msaf052_Supplementary_Data

## Data Availability

All genomic data generated in this project have been submitted to NCBI under BioProject, accession number PRJNA1161641.
