# Supplementary material for: Genomic Insights into the Successful Invasion of the Avian Vampire Fly (Philornis downsi ) in the Galápagos Islands
Source: Mol Biol Evol. 2025 Mar 28;42(3):msaf052. doi: 10.1093/molbev/msaf052 (PMC11951964; doi:10.1093/molbev/msaf052)
Supplement: msaf052_Supplementary_Data [file msaf052_supplementary_data.zip › Supplementary.pdf]

Supplementary Materials

A

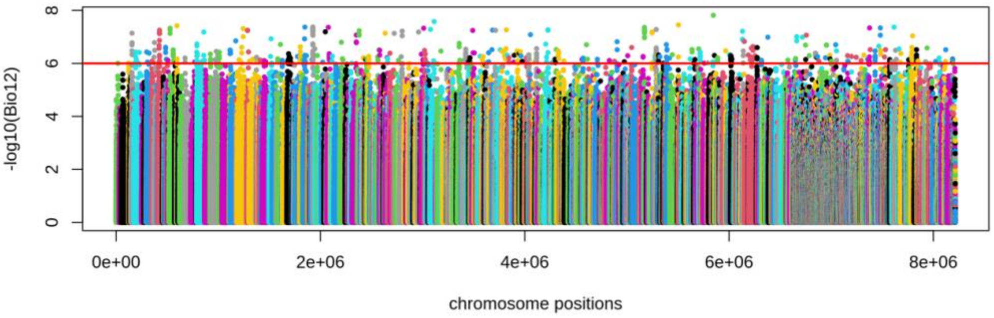

B

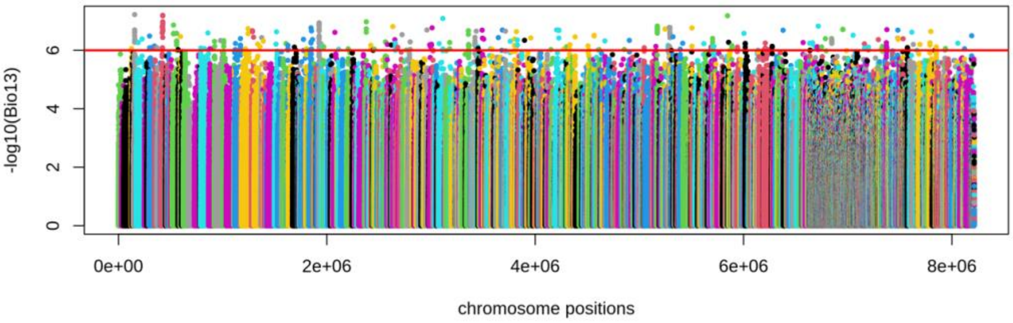

C

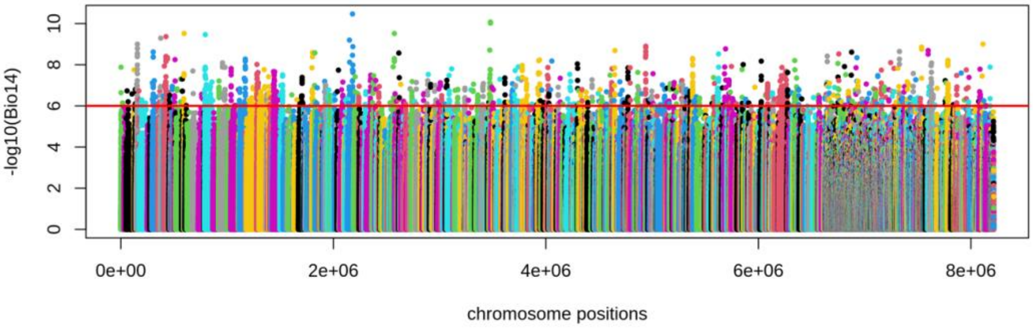

Supplementary Fig. 1. Inference of Gene-Environment Associations. Manhattan plots showing the distribution of significance values $-\log_{10}$(adjusted p-value) obtained by LFMM showing the genome-wide association of SNPs for particular environmental variable, **(A)** Annual precipitation (BIO12) **(B)** Precipitation of Wettest Month (BIO13) and **(C)** Precipitation of driest Month (BIO14).

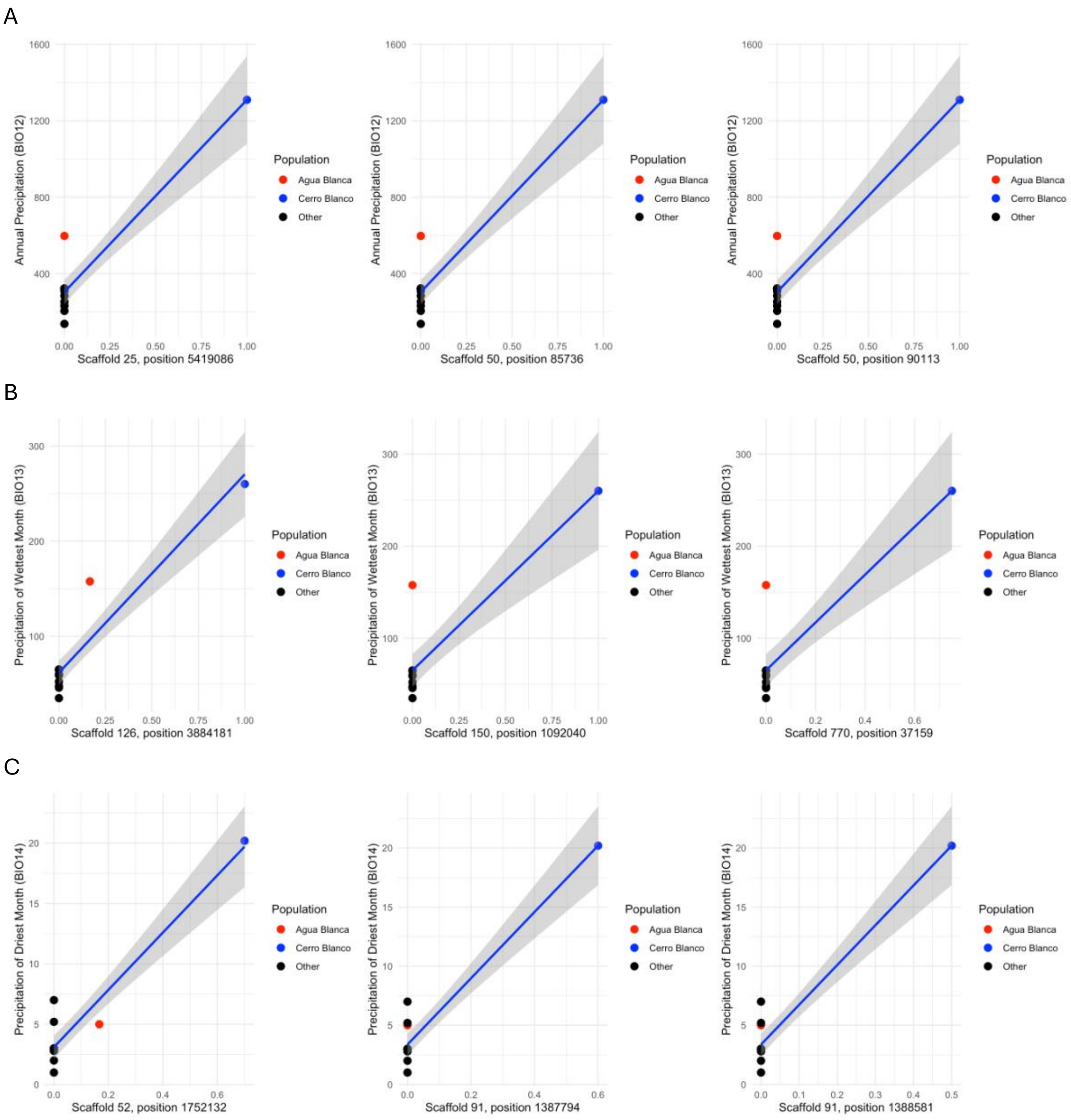

Supplementary Fig. 2. Correlation of population allele frequency of top candidate SNPs that are significantly associated with three precipitation-related bioclimatic variables **(A)** Annual precipitation (BIO12) **(B)** Precipitation of Wettest Month (BIO13) and **(C)** Precipitation of driest Month (BIO14).

Supplementary Table 1 to 7

Supplementary Table 1: Details on the sample used in this study

Sample_ID	Collected in	Field Code	Sex	Locality
P_08_60	2021	ABDNN #08 - Fly 60	Female	Agua Blanca
P_08_63	2021	ABDNN #08 - Fly 63	Female	Agua Blanca
P_08_64	2021	ABDNN #08 - Fly 64	Female	Agua Blanca
CB_B3_B	2015	GF.B3.69CB2015	Female	Cerro Blanco
CB_B4_B	2015	GF.B4.69.CB2015	Unknown	Cerro Blanco
CB_B5_B	2015	GF.B5.69CB2015	Female	Cerro Blanco
CB_B6_B	2015	GF.B6.69CB2015	Female	Cerro Blanco
CB_C3_B	2015	GF.C3.69CB2015	Female	Cerro Blanco
CB_C4_B	2015	GF.C4.69CB2015	Female	Cerro Blanco
CB_C5_B	2015	GF.C5.69CB2015	Female	Cerro Blanco
CB_E3	2015	GF.E3.69CB2015	Male	Cerro Blanco
CB_E5	2015	GF.E5.69CB2015	Male	Cerro Blanco
CB_E7	2015	GF.E7.69CB2015	Male	Cerro Blanco
CP14_01	2017	JDF1	Female	Daphne Mayor
CP12_1	2017	JDF2	Female	Daphne Mayor
CP12_2	2017	JDF3	Female	Daphne Mayor
IS_G5_B	2016	ISACURA5.G5	Female	Isabela
IS_G6	2016	Isabela.G6.Fly1	Female	Isabela
IS_G7_B	2016	Isabela.G7.Fly1	Female	Isabela
IS_G8_2	2016	Isabela.G8.Fly2	Male	Isabela
IS_G8_B	2016	Isabela.G8.Fly1	Male	Isabela
IS_G9_B	2016	Isabela.G9.Fly1	Male	Isabela
IS_H1_B	2016	Isabela.H1.Fly1	Male	Isabela
IS_H2_B	2016	Isabela.H2.Fly1	Female	Isabela
IS_H3	2016	Isabela.H3.	Male	Isabela
IS_H6	2016	Isabela.H6.Fly1	Unknown	Isabela
CP14_10	2021	JMF1	Female	Marchena
CP14_11	2021	JMF2	Female	Marchena
CP11_11	2021	JMF3	Female	Marchena
CP14_12	2021	JMM5	Male	Marchena
CP11_13	2021	JMM6	Male	Marchena
CP14_13	2021	JMM7	Male	Marchena
CP14_14	2021	JMM8	Male	Marchena
CP14_15	2021	JMM9	Male	Marchena
CP12_7	2021	JMM10	Male	Marchena
CP12_8	2021	JMM11	Male	Marchena
PZ_C2_B	2019	JK2019-60	Female	Pinzon
PZ_C7_B	2019	JK2019-08	Male	Pinzon
PZ_D1_B	2019	JK2019-03	Male	Pinzon
PZ_D2	2019	JK2019-16	Male	Pinzon
PZ_D4_B	2019	JK2019-13	Male	Pinzon
PZ_D5	2019	JK2019-26	Female	Pinzon

PZ_D9	2019	JK2019-37	Female	Pinzon
PZ_E1	2019	JK2019-40	Female	Pinzon
PZ_E3	2019	JK2019-12	Male	Pinzon
PZ_E5	2019	JK2019-15	Male	Pinzon
CP14_16	2019	JSCF1	Female	Santa Cruz
CP13_2	2019	JSCF2	Female	Santa Cruz
CP14_17	2019	JSCF3	Female	Santa Cruz
CP13_5	2019	JSCF5	Female	Santa Cruz
CP14_18	2019	JSCF6	Female	Santa Cruz
CP13_8	2022	JSCM2	Male	Santa Cruz
CP13_9	2022	JSCM3	Male	Santa Cruz
CP13_10	2022	JSCM4	Male	Santa Cruz
CP13_11	2022	JSCM5	Male	Santa Cruz
CP13_12	2022	JSCM6	Male	Santa Cruz
CP09_1	2020	JSM1	Male	Santiago
CP14_02	2020	JSM2	Male	Santiago
CP14_03	2020	JSM3	Male	Santiago
CP14_04	2020	JSM4	Male	Santiago
CP14_05	2020	JSM5	Male	Santiago
CP14_06	2020	JSF16	Female	Santiago
CP14_07	2020	JSF17	Female	Santiago
CP14_08	2020	JSF18	Female	Santiago
CP11_1	2020	JSF19	Female	Santiago
CP14_09	2020	JSF20	Female	Santiago

Supplementary Table 2: Genome-wide average Nucleotide diversity (pi) among mainland and Island Populations.

**Population**	**Location type**	**Genome-wide average Pi**
Cerro Blanco	Mainland	0.00343439
Isabela	Island	0.0023039
Marchena	Island	0.00218174
Pinzon	Island	0.00239485
Santa Cruz	Island	0.00238387
Santiago	Island	0.00235241

Supplementary Table 3: Genome-wide average Tajima’s D among mainland and Island Populations.

**Population**	**Location type**	**Genome-wide average Tajima’s D**
Cerro Blanco	Mainland	0.889554
Isabela	Island	1.00999
Marchena	Island	0.945113
Pinzon	Island	0.971654
Santa Cruz	Island	0.970577
Santiago	Island	0.914032

Supplementary Table 4: CV errors for Admixture run from K=1 to 10. Lower CV errors correspond to the more likely structure of the data, in this case, K=2 and 3.

K value	CV error
1	0.49
2	0.39
3	0.39
4	0.41
5	0.42
6	0.48
7	0.49
8	0.52
9	0.53
10	0.57

Suppelementary Table 5: ABBA-BABA statistics. P1/P2 - two island populations of *P. downsi* representing sister groups; P3 – third island populations of *P. downsi*. Cerra Blanco population from the mainland was used as an outgroup. Two significant evidence of gene flow are highlighted.

P1	P2	P3	D stats	Z-score	p-value	f4-ratio	BBAA	ABBA	BABA
DaphneMayor	Isabela	Marchena	0.003	0.64	0.52	0.02	491,020	484,960	481,620
Isabela	DaphneMayor	Pinzon	0.002	0.45	0.65	0.03	495,400	485,790	483,946
Isabela	DaphneMayor	SantaCruz	0.007	1.30	0.19	0.11	502,079	486,059	479,734
DaphneMayor	Isabela	Santiago	0.012	2.11	0.03	0.15	496,603	487,817	476,678
Marchena	DaphneMayor	Pinzon	0.010	2.08	0.04	0.17	492,752	492,541	482,325
Marchena	DaphneMayor	SantaCruz	0.014	2.35	0.02	0.20	499,520	492,900	479,555
Marchena	DaphneMayor	Santiago	0.003	0.48	0.63	0.04	496,644	486,119	483,482
**Pinzon**	**DaphneMayor**	**SantaCruz**	**0.012**	**3.21**	**0.00**	**0.18**	**499,112**	**492,703**	**481,255**
Pinzon	DaphneMayor	Santiago	0.004	0.93	0.35	0.05	497,293	486,979	483,281
SantaCruz	DaphneMayor	Santiago	0.011	2.96	0.00	0.13	495,505	491,600	480,674
Marchena	Isabela	Pinzon	0.009	1.62	0.11	0.14	494,697	489,302	480,930
Marchena	Isabela	SantaCruz	0.007	1.03	0.30	0.11	502,575	486,289	479,270
Marchena	Isabela	Santiago	0.014	2.43	0.02	0.18	492,861	490,135	476,359
Pinzon	Isabela	SantaCruz	0.005	1.39	0.17	0.08	499,627	488,737	483,614
**Santiago**	**Isabela**	**Pinzon**	**0.013**	**3.37**	**0.00**	**0.19**	**493,733**	**491,064**	**478,896**
Santiago	Isabela	SantaCruz	0.009	2.09	0.04	0.13	499,815	486,256	477,750
Marchena	Pinzon	SantaCruz	0.002	0.38	0.70	0.03	498,016	490,376	488,478
Pinzon	Marchena	Santiago	0.001	0.23	0.82	0.01	491,818	489,083	488,022
Santiago	Marchena	SantaCruz	0.002	0.35	0.73	0.02	494,943	488,140	486,654
Santiago	Pinzon	SantaCruz	0.003	0.98	0.33	0.05	493,543	489,699	486,315

Supplementary Table 6: Candidate genomic regions showing strong genetic divergence between mainland and island populations of *P. downsi*. The strongest genetic divergence between mainland and island populations was a 15kb window (Scaffold 54:3,270,001-3,285,000) with a ZF_{ST} of 6.73 is highlighted.

Scaffold id	Scaffold length (bp)	Position start (bp)	Position end (bp)	Total SNPs in the window	Mean F_{ST}	ZF_{ST}
54	5,278,662	225,001	240,000	29	0.61	5.22
54	5,278,662	285,001	300,000	173	0.6	5.09
54	5,278,662	1,650,001	1,665,000	148	0.63	5.5
**54**	**5,278,662**	**3,270,001**	**3,285,000**	**184**	**0.72**	**6.73**
54	5,278,662	3,405,001	3,420,000	181	0.64	5.56
54	5,278,662	3,435,001	3,450,000	213	0.67	6.07
262	1,562,575	1	15,000	95	0.63	5.45
262	1,562,575	45,001	60,000	97	0.63	5.5
424	2,625,841	630,001	645,000	221	0.65	5.78
424	2,625,841	870,001	885,000	175	0.61	5.15
440	1,495,077	300,001	315,000	189	0.6	5.09
514	2,503,269	1,215,001	1,230,000	83	0.65	5.71
611	912,942	840,001	855,000	42	0.64	5.58
716	5,374,294	1,545,001	1,560,000	261	0.64	5.69
784	2,096,348	435,001	450,000	282	0.62	5.38
784	2,096,348	720,001	735,000	112	0.62	5.4

Supplementary Table 7: 19 bioclimatic variables from the WorldClim database associated with temperature and precipitation measurements from mainland (Ecuador) and Galapagos Island locations used in this study.

**Variable**	**Variable description**	**Agua Blanca**	**Cerro Blanco**	**Daphne Mayor**	**Isabela**	**Marchena**	**Santa Cruz**	**Santiago**	**Pinzon**
BIO1(°C)	Annual Mean Temperature	22.83	23.35	24.36	21.48	24.54	23.31	24.33	22.65
BIO2 (°C)	Mean Diurnal Range (Mean of monthly (max temp - min temp))	9.14	9.19	7.37	9.47	8.87	8.30	9.00	8.12
BIO3	Isothermality (BIO2/BIO7) (×100)	75.10	83.40	61.39	72.26	68.95	62.62	68.26	62.58
BIO4	Temperature Seasonality (standard deviation ×100)	108.49	63.24	164.70	126.08	140.96	174.94	149.57	170.63
BIO5 (°C)	Max Temperature of Warmest Month	28.70	28.82	30.50	28.10	31.17	30.10	31.04	29.36
BIO6 (°C)	Min Temperature of Coldest Month	16.53	17.76	18.50	15.00	18.30	16.84	17.86	16.38
BIO7(°C)	Temperature Annual Range (BIO5-BIO6)	12.17	11.06	12.00	13.10	12.87	13.26	13.18	12.98
BIO8 (°C)	Mean Temperature of Wettest Quarter	24.17	24.03	26.20	22.80	26.07	25.28	26.29	24.53
BIO9 (°C)	Mean Temperature of Driest Quarter	21.75	22.75	22.77	20.27	23.73	21.47	22.80	21.42
BIO10 (°C)	Mean Temperature of Warmest Quarter	24.21	24.14	26.52	23.15	26.37	25.57	26.29	24.90

BIO11 (°C)	Mean Temperature of Coldest Quarter	21.63	22.61	22.42	20.00	22.87	21.29	22.58	20.68
BIO12 (mm)	Annual Precipitation	597.67	1310.40	137.00	309.00	296.67	252.80	231.80	205.20
BIO13 (mm)	Precipitation of Wettest Month	157.67	260.00	35.00	60.00	61.00	52.40	47.60	46.00
BIO14 (mm)	Precipitation of Driest Month	5.00	20.20	1.00	7.00	2.33	5.20	2.80	2.80
BIO15	Precipitation Seasonality (Coefficient of Variation)	22.83	23.35	24.36	21.48	24.54	23.31	24.33	22.65
BIO16 (mm)	Precipitation of Wettest Quarter	22.83	23.35	24.36	21.48	24.54	23.31	24.33	22.65
BIO17(mm)	Precipitation of Driest Quarter	22.83	23.35	24.36	21.48	24.54	23.31	24.33	22.65
BIO18 (mm)	Precipitation of Warmest Quarter	22.83	23.35	24.36	21.48	24.54	23.31	24.33	22.65
BIO19 (mm)	Precipitation of Coldest Quarter	22.83	23.35	24.36	21.48	24.54	23.31	24.33	22.65
